# Community-Engaged Development, Implementation, and Evaluation of an Interprofessional Education Workshop on Gender-Affirming Care

**DOI:** 10.1089/trgh.2019.0036

**Published:** 2019-10-25

**Authors:** M. Kathryn Allison, S. Alexandra Marshall, Dani Smith Archie, Taylor Neher, Gray Stewart, Michael E. Anders, M. Kathryn Stewart

**Affiliations:** ^1^ Department of Health Behavior & Health Education, Fay W. Boozman College of Public Health, University of Arkansas for Medical Sciences, Little Rock, Arkansas.; ^2^ Arkansas Transgender Equality Coalition, Little Rock, Arkansas.; ^3^ Division of Academic Affairs, Office of Educational Development, University of Arkansas for Medical Sciences, Little Rock, AR.; ^4^ Department of Health Policy & Management, Fay W. Boozman College of Public Health, University of Arkansas for Medical Sciences, Little Rock, Arkansas.

**Keywords:** transgender, interprofessional education, gender affirming care

## Abstract

**Background:** Transgender/nonbinary (trans/NB) patients face stigma in health care settings. Health care professionals' training on trans/NB issues has historically been lacking. Interprofessional education (IPE) provides an opportunity to improve knowledge and attitudes across health care professions. The purpose of this study was to: (a) describe the development and implementation of an IPE workshop on gender-affirming care through a trans/NB community-academic partnership and (b) examine the impact of the workshop on student knowledge and attitudes.

**Methods:** The workshop included a slide presentation on basic terminology and concepts, video clips of trans/NB patient**–**provider interactions, facilitated discussions of affirming practices, and a trans/NB panel. Nonparametric statistical analysis of pre- and post-survey data from 58 workshop participants measured changes in student knowledge and attitudes.

**Findings:** Students demonstrated statistically significant improvements in knowledge (*t*=−12.72; *p*<0.01) and interpersonal comfort (*t*=−2.06; *p*<0.05) as well as sex and gender beliefs (*t*=−3.06; *p*<0.05) on subscales from the Transgender Attitudes & Beliefs Scale. The results demonstrated no differences on the human value subscale (*t*=−0.69; *p*=0.49) or on health care professional questions (*t*=−1.23; *p*=0.23).

**Conclusions:** A community-academic partnership developed and implemented this brief interactive educational intervention, which can improve both knowledge and attitudes about trans/NB individuals' health among health professional students.

## Introduction

Transgender and nonbinary (trans/NB) individuals face unique obstacles to accessing quality health care in the United States, including many who regularly face discrimination in health care encounters due to their trans/NB status.^1–3^ Trans/NB patients report health care providers who display stigmatizing attitudes and behaviors, such as the use of harsh language or verbal harassment, inappropriate use of names or pronouns, insensitivity toward patients' expressed genders, general displays of discomfort, forcing unwanted or unneeded care, deferral of treatment, blaming patients for their own health, or outright denying necessary treatment.^4,5^ Such experiences and anticipation of further discrimination in health care settings often lead trans/NB patients to delay seeking care,^6,7^ which can result in adverse health outcomes.^8,9^

Nine out of 10 trans/NB individuals believe that there are not enough health care professionals who are adequately trained to address their unique health care needs.^5^ Half of trans/NB patients report having to teach their providers about trans/NB health, a factor associated with a four-fold increase in likelihood of delaying future care.^1,2,10,11^ This perceived shortage of providers trained to meet the unique health care needs of trans/NB patients creates an additional barrier to health care access.^10,12^

Although providers often lack the knowledge and skills necessary to treat lesbian, gay, bisexual, trans/NB, and queer/questioning (LGBTQ) patients for a range of health care issues; this is particularly true for trans/NB individuals.^13,14^ Previous research found that physicians had insufficient knowledge of trans/NB health and were unsure where to access reliable information on the topic.^14^ Pharmacy students in residency have been shown to feel similarly ill-prepared to serve trans/NB patients.^15^ Providers also struggle to identify and make referrals to other providers who are more competent to care for their trans/NB patients.^8,14^ Providers' lack of knowledge of trans/NB-specific treatments and resources can hinder their abilities to gather salient information about patients' specific health needs and refer them to specialized care, thereby further limiting patients' access to needed health care services.^16^

This common knowledge gap stems from the omission of LGBTQ-specific education throughout all levels of professional training.^17^ More than half of providers report insufficient training in trans/NB-specific care and exposure to trans/NB patients as barriers to their ability to provide appropriate care to these patients.^18^ A survey of medical schools in the United States and Canada found that the average time dedicated to LGBTQ-related content in medical education was 5 h, with most reporting no LGBTQ-specific instruction during third- and fourth-year clinical rotations when students encounter patients in a range of medical specialties.^19^ Thus, medical students have a considerable underexposure to both LGBTQ-specific content and diverse LGBTQ patients, particularly trans/NB patients.^15,19^

Providing evidence-based information in medical education challenging the foundational elements of stigma can reduce the prevalence of stigma among medical professionals; however, contact with those stigmatized is the most effective strategy for reducing stereotyping and discriminatory behavior among providers.^5,20^ Exposure to members of the trans/NB population as both real and simulated patients in clinical training increases the likelihood that physicians will have positive attitudes toward these patients later in their careers^10^ and that students will develop the communication skills that are necessary for providing effective and respectful care to this population.^10,21^ Increased contact with diverse LGBTQ individuals in combination with education on trans/NB-related health topics results in more positive attitudes toward this population and a more complete understanding of their unique needs.^5,10,21^ Hence, educational interventions designed to increase health care providers' cultural competence should address knowledge as well as provide direct contact with trans/NB patients.

Evidence demonstrates that brief educational interventions can improve providers' knowledge and attitudes related to trans/NB health and health care.^22–24^ However, relevant training often emphasizes lesbian, gay, and bisexual (LGB) health, with limited information specific to trans/NB populations. Therefore, students have a greater understanding of LGB health compared with that of the trans/NB community, and they feel more comfortable treating cisgender LGB patients than trans/NB or intersex patients.^25^ To address this gap, training for future health care providers should include trans/NB-specific content and interaction with trans/NB patients. Educational interventions should involve a basic understanding of sexual orientation and gender identity, along with best medical practices within gender-affirming care and basic hormone knowledge.^26^ To ensure safety of care and promote an inclusive health care environment, all medical professionals, regardless of future specialties, should receive this training as a core competency.^26^

Interprofessional education (IPE) offers an opportunity to engage a range of future health care professionals in interactive discussion and learning on trans/NB health and health care. IPE involves students from two or more health professions learning together during their professional training with the objective of cultivating collaborative practice for patient-centered care.^27^ Emerging evidence demonstrates that IPE can change attitudes and enable collaborative practice.^28^ Educators can use a number of formats that are focused on a wide range of health topics, while allowing individuals to develop a sense of belonging in a team, autonomy, and competence in developing patient care plans.^28^ Hence, IPE offers an opportunity to engage multiple health professions in discussion about collaborative, patient-centered care for trans/NB patients. This article describes the development and implementation of a community co-led IPE workshop on gender-affirming care and documents changes in student knowledge and attitudes.

## Methods

### Ethical considerations

The authors' university Institutional Review Board (IRB) determined this study as exempt from human subjects research review.

### Setting

This study took place at an academic health center in a Southern state. With five colleges (Medicine, Pharmacy, Nursing, Health Professions, and Public Health) and a Graduate School, the university enrolls more than 2700 students. The university has a strong IPE initiative and offers competitive intramural grants for program development. An interprofessional team, including public health researchers, health care and mental health care providers, trans/NB individuals, the director of the Center for Patient and Family Centered Care, and the director of the Simulation Center, received one of these grants to develop an IPE activity for medical, nursing, pharmacy, public health, allied health, and graduate students. This interprofessional team, in collaboration with the trans/NB community, developed a 2-h IPE workshop on gender-affirming care.

### Community partnership

In 2015, the Arkansas Transgender Equality Coalition (ArTEC) and the university's college of public health formed a partnership to develop the Transform Health Arkansas Initiative. The purpose of the Transform Health Arkansas Initiative was to engage trans/NB individuals across the state in defining their health-related research interests and priorities.^29,30^ Through a statewide survey and summits supported by Transform Health across the state, trans/NB Arkansans identified the need for provider education on trans/NB health as a top health-related priority. The project described herein grew out of collaboration between trans/NB individuals, researchers, and patient advocates focused on addressing this prioritized concern.

One of the investigators, a psychotherapist and member of the trans/NB community, engaged the Trans/NB Patient Advisory Board to inform the workshop structure and content. The Trans/NB Patient Advisory Board elected to develop scripts depicting scenarios based on actual health care experiences of trans/NB community members for videos, which simulated trans/NB patients' interactions with providers and other staff in health care settings. In addition, a co-investigator conducted semi-structured qualitative interviews with two health care providers practicing gender-affirming care. These providers offered insights on facilitators of and barriers to gender-affirming care, and their feedback informed the work of the Trans/NB Patient Advisory Board.

### Scenario/script development

A small group of trans/NB community members brainstormed incidents that either they or a trans/NB acquaintance of theirs had experienced in a health care setting. The investigators then grouped these experiences into broader issue categories (e.g., maintenance of confidentiality, inappropriate questions or comments, misgendering, and dealing with discrepancies across identity documentation) and combined them into scenes to address multiple issues in each scene. The investigators, in collaboration with trans/NB community members, then developed a script for each scene.

### Video production

Filming locations were the university Simulation Center, featuring realistic clinical settings, and a real community pharmacy. Actual trans/NB community members played the roles of trans/NB patients, whereas students and paid actors played the roles of health care professionals and staff. The scenarios included both positive and negative patient interactions with front desk staff, nurses, physicians, medical students, and a pharmacist. The edited film strategically placed questions between scenes to prompt interprofessional discussions during the workshop.

### Additional content

Before the video, an investigator delivered a PowerPoint presentation, with information on terminology, health disparities, and affirming care practices. Definitions included “gender identity,” “gender expression,” “biological sex,” “sexual orientation,” “transgender,” “gender non-binary,” and “cisgender.” Discussion topics were: (a) four components of identity (body, mind, appearance, and attraction), (b) the transitioning process, (c) health disparities that trans/NB individuals commonly experience, and (d) unique health needs of trans/NB patients. The presentation highlighted state-specific results of the U.S. Trans Survey^1^ related to employment, education, health care, and the effects of discrimination on mental health. The presentation then included examples of inclusive, affirming health care practices aligned with principles of patient-centered care, as well as terms and phrases to avoid when talking to a trans/NB person. To conclude the workshop, a panel of trans/NB patients facilitated a question and answer session.

### Assessment tools

Pre- and post-questionnaires examined students' knowledge of trans/NB terminology, health, and health care practice, as well as attitudes toward trans/NB individuals and gender-affirming care. The assessment of knowledge about the content contained 10 questions: 5 fill-in-the-blank questions assessing understanding of basic terminology, 3 true or false questions assessing knowledge of trans/NB health topics, and 2 multiple choice questions assessing knowledge of gender-affirming care practices. The *Transgender Attitudes and Beliefs Scale (TABS)*^31,32^—a validated scale assessing interpersonal comfort, sex/gender beliefs, and human value—elicited the students' attitudes. This assessment consisted of 29 questions measuring attitudes toward trans/NB individuals, trans/NB health, and gender-affirming care by using a five-point Likert ordinal scale (i.e., strongly agree, somewhat agree, neither/neutral/unsure, somewhat disagree, and strongly disagree). The TABS instrument improved on previous instruments that failed to take into account the religious climate of the population and accommodate beliefs held by many evangelical Christians that human beings have intrinsic value, regardless of their identity or behavior. In addition, testing of TABS occurred in a more generalizable population than previous measures, it incorporates more current civil rights issues for trans/NB people, and it is shorter than existing multidimensional transgender attitude measures.^32^ We included three additional questions to assess the students' attitudes toward trans/NB patients, their responsibility as health professionals to serve trans/NB patients, and their concerns about cisgender patients knowing that they serve trans/NB patients. We refer to this attitude subscale as “healthcare professionals.”

### Workshop pilot and implementation

We piloted the workshop with 10 public health graduate students in the university's college of public health course on racial and ethnic health disparities in the spring of 2018, in preparation for their service-learning project focusing on trans/NB health care experiences. After this pilot, we implemented the full workshop in the summer of 2018 as part of an interprofessional course on patient- and family-centered care offered to students in a variety of disciplines through a partnership with the university's Office of Interprofessional Education. Faculty from the university's college of public health and members of the trans/NB community involved in the Transform Health Arkansas Initiative co-taught the gender-affirming care workshop on the first day of the course. Fifty-eight students studying health professions or nursing were enrolled in the course. To assess longer-term impact, after 9 months the investigators attempted a follow-up survey of workshop participants, but the response rate was too low for analysis.

### Procedures and analysis

Immediately before and after the IPE workshop on gender-affirming care, participants completed pre- and post-questionnaires assessing knowledge and attitudes. Repeated-measures *t*-tests were used to determine whether the intervention had an effect on knowledge and attitudes.

This article was jointly authored by allies (M.K.A., S.A.M., T.N., M.E.A., and M.K.S.) and trans/NB individuals (D.S.A. and G.S.), all of whom were a part of the collaborative project described.

## Results

Fifty-eight students participated in the workshop. Of these, 56 students completed both the pre- and post-knowledge survey, 51 completed both the pre- and post-interpersonal comfort attitude subscales, and 50 completed both the remaining pre- and post-attitudes subscales. See Table 1 for means and standard deviations for the pre- and post-knowledge and attitude scales.

**Table 1.** Descriptive Statistics and *t*-Test Results for Knowledge and Attitude Scores

Outcome	Pre-test		Post-test		*n*	95% CI for mean difference	*r*	*t*	df
	*M*	SD	*M*	SD					
Knowledge	5.20	2.06	9.29	1.06	56	−4.73 to −3.44	−0.10	−12.72^*^	55
IC	58.43	9.81	61.75	8.54	51	−6.47 to −0.08	0.24	−2.06^*^	50
SGB	32.80	9.92	38.78	9.50	50	−9.91 to −2.05	−0.02	−3.06^*^	49
HV	24.34	1.36	24.56	1.66	50	−0.86 to 0.42	−0.10	−0.69	49
HP	13.62	1.24	13.90	1.37	50	−0.74 to 0.18	0.24	−1.23	49

^*^ *p*<0.05.

CI, confidence interval; HP, healthcare professionals; HV, human value; IC, interpersonal comfort; SBG, sex/gender beliefs; SD, standard deviation.

Table 1 also presents results of repeated-measures *t-*tests, which examined whether students demonstrated improvements in knowledge and attitudes after participating in the intervention. Students exhibited statistically significant improvements in knowledge (*t*=−12.72; *p*<0.01) and on the interpersonal comfort (*t*=−2.06; *p*<0.05) and sex and gender beliefs (*t*=−3.06; *p*<0.05) subscales of the TABS. No differences were found on the human value (*t*=−0.69; *p*=0.49) subscale or health care professional (*t*=−1.23; *p*=0.23) questions. Changes in knowledge were not significantly associated with attitude changes in this sample (data not shown).

## Discussion

Members of the Transform Health Arkansas partnership developed this 2-h IPE workshop curriculum to address the community-identified need for provider education on trans/NB health.^29,30^ The workshop included a content lecture, videos of case-based scenarios with facilitated small-group discussions, and direct exposure through a panel discussion by trans/NB individuals. Analysis of pre-/post-workshop evaluation data showed improvements in students' knowledge and attitudes on two of the three subscales measuring attitudes about trans/NB patients. Although we do not have longer-term follow-up data to determine the extent to which the students maintained these improvements, our findings suggest that even a brief workshop intervention such as this can have a worthwhile, positive impact.

The context of this work in the southern state of Arkansas is relevant because multiple studies have shown that the prevalence of negative health care experiences and discrimination in housing, employment, and education are all higher for trans/NB people living in the South.^1,6^ These issues are particularly pressing in states such as Arkansas where socially conservative beliefs and religiosity are among the highest in the country^33–36^ and rates of psychosocial stress among trans/NB people are also higher than in other regions.^37^ Our workshop incorporated known trans-sensitive stigma- and bias-reduction strategies, including education on stigma and bias and their impact, the use of participatory methods, and direct and indirect contact, counter-stereotyping and stereotype replacement, individuation, and perspective-taking.^13,38,39^ Specifically, the workshop was developed jointly with trans/NB individuals and academic faculty through a community-academic partnership. The workshop included content on stigma against trans people and how it increases disparities, as well as exposure to trans/NB individuals through small-group discussions about videotaped case scenarios and an in-person panel discussion.

We found the greatest changes in the knowledge measures and the sex and gender beliefs attitude subscale. Although our sample size was likely insufficient to detect a statistically significant association between knowledge and attitudes, our results may suggest that greater knowledge about trans/NB people can have a positive impact on beliefs about sex and gender. We documented slight increases in interpersonal comfort, but the mean change in the human value subscale was not significant. The baseline responses for items on both of these subscales were relatively high, particularly for human value, which had a mean of 4.86 on a five-point scale, leaving little room for improvement.

Our finding of high baseline responses related to human value is similar to those of Kanamori et al.,^31^ which were based on data from a sample in which 42% had religious roots in evangelical Christianity. They interpret their findings as suggesting that “evangelical Christians firmly hold to the intrinsic value of the person, though their ratings are lower on matters of transgender civil rights [measured by the sex and gender beliefs subscale] and the degree of comfort in associating with transgender individuals” (p. 1513).^31^ We did not collect data on the religious beliefs of our participants and, therefore, cannot determine whether this dynamic played a role, but it is worth noting that 70% of Arkansans identify as “highly religious” and 46% of the state's population identify as evangelical Christian, the fifth highest in the nation.^33^

Others have implemented brief educational interventions in health professional training settings. For example, in their evaluation of two mandatory 1-h lectures for medical students, Turban et al. observed knowledge improvements related to gender dysphoria and hormone therapy and, similar to our results, found no association between knowledge gain and attitudes regarding the ethics of hormone therapy.^40^ Braun et al. documented increased knowledge in most domains they studied, as well as reduced transphobia after a 10-session lunch elective course on transgender health with students from a variety of health professions.^22^ In another study, third-year pharmacy students participating in a 2-h lecture showed higher mean scores in knowledge and confidence caring for trans/NB patients compared with fourth-year students who had not had this exposure.^24^ Strong and Folse also showed significant improvement in knowledge and attitudes about LGBTQ patients among nursing students after a 1-h educational session.^41^ After three 2-h transgender-health training sessions, Lelutiu-Weinberger et al. reported a reduction in negative attitudes toward transgender patients among clinicians at an urban medical clinic.^23^ Our data are consistent with these studies in showing knowledge and attitude changes regarding trans/NB health after even a relatively brief educational intervention.

### Limitations

The small convenience sample of participants in this workshop limits the generalizability of our results, but the significant changes in knowledge and attitudes documented are promising. The workshop was embedded within a course on Patient and Family Centered Care, so it is possible that the population of students electing to participate were more sensitive to issues related to patient-centered care. The use of self-reported data may have introduced a response bias, with students providing less truthful, more socially acceptable responses, but the baseline responses for several outcomes indicate that this was not a problem. In addition, the data we collected immediately before and after the workshop may not reflect long-term results or future clinical behaviors. No data are available on participants' perceptions of what aspects of the workshop were most impactful, but in our opinion, those aspects with direct involvement of trans/NB individuals (i.e., the videos and the panel) were essential.

## Conclusion

Our findings suggest that this brief, interactive educational intervention, developed and implemented through an academic partnership with trans/NB individuals, holds promise for improving both knowledge about and attitudes toward trans/NB individuals. Future efforts include offering this workshop to health care practitioners as a continuing education credit. Further research is needed to assess this intervention's long-term impact on knowledge and attitude changes in a broader audience of health professional students and to determine whether such changes translate to improved trans/NB health care and health outcomes.

## Abbreviations Used

CI: confidence interval
HP: healthcare professionals
HV: human value
IC: interpersonal comfort
IPE: interprofessional education
LGB: lesbian, gay, and bisexual
LGBTQ: lesbian, gay, bisexual, trans/NB, and queer/questioning
SBG: sex/gender beliefs
SD: standard deviation
TABS: Transgender Attitudes and Beliefs Scale
trans/NB: transgender/nonbinary
